# Immunotherapy combined with chemotherapy improved clinical outcomes over bevacizumab combined with chemotherapy as first‐line therapy in adenocarcinoma patients

**DOI:** 10.1002/cam4.5356

**Published:** 2022-10-21

**Authors:** Min Wang, Ji Li, Shuhui Xu, Yuying Li, Jiatong Li, Jinming Yu, Xiaoyong Tang, Hui Zhu

**Affiliations:** ^1^ Department of Radiation Oncology Shandong Cancer Hospital and Institute Shandong First Medical University and Shandong Academy of Medical Sciences Jinan Shandong China; ^2^ Department of Radiation Oncology Shandong Cancer Hospital and Institute affiliated of Shandong University Jinan Shandong China

**Keywords:** adenocarcinoma, bevacizumab, first‐line therapy, immunotherapy, NSCLC

## Abstract

**Purpose:**

No definite conclusion has yet to be reached for the first‐line treatment combined with chemotherapy for advanced adenocarcinoma NSCLC patients with negative driver genes. This study sought to compare the clinical outcomes of Beva+ChT and IO+ChT as first‐line treatment for this population and investigated whether the statuses of BM, PD‐L1 expression, and KRAS and TP53 mutations could influence the results.

**Patients and methods:**

The clinical data of patients with adenocarcinoma NSCLC who received first‐line therapy were retrospectively collected and the patients were assigned to the IO+ChT and Beva+ChT groups. The disease control rate (DCR), progression‐free survival (PFS), and overall survival (OS) were evaluated between the two groups. The survival effects of BM, PD‐L1 expression, and KRAS and TP53 mutations were also evaluated.

**Results:**

From April 2018 to October 2020, a total of 105 patients with first‐line therapy were included in our analysis; 54 (51.4%) patients were included in the IO+ChT group and 51 (48.6%) patients were included in the Beva+ChT group. The results showed that OS (NR vs. 18.3 m, *p* = 0.011) and PFS (14.9 m vs. 6.3 m, *p <* 0.001) were superior in patients in the IO+ChT group than in patients in the Beva+ChT group. Further analysis revealed that the OS (median OS: NR vs. 14.7 months, *p* = 0.039) and PFS (median PFS: 18.5 vs. 5.5 months, *p* < 0.001) advantages of the IO+ChT group were also seen in the PD‐L1 > 1% subgroup but were not seen in the PD‐L1 < 1%, BM or KRAS mutation subgroups.

**Conclusions:**

ICIs combined with ChT improved clinical outcomes over Beva combined with ChT as first‐line therapy for adenocarcinoma patients without driver gene alterations, especially in patients with PD‐L1 ≥ 1%.

## Graphical abstract

ICIs combined with ChT improved clinical outcomes over Beva combined with ChT as first‐line therapy in adenocarcinoma patients without driver‐gene alterations, especially in patients with PD‐L1≥1%.

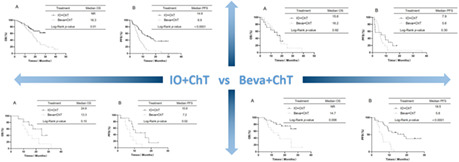

## 1 INTRODUCTION

Worldwide, lung cancer remains the most common type of cancer and has a high morbidity rate.^1^ For decades, platinum‐based chemotherapy has been the standard first‐line treatment for advanced non‐small‐cell lung cancer (NSCLC) without a driver gene mutation.^2^ The addition of bevacizumab to chemotherapy extended survival to more than 1 year in this population (median OS, 12.5 months) for the first time in the early global phase III ECOG 4599 study.^3^ Additionally, the European phase III randomized controlled trial AVAil showed that bevacizumab plus chemotherapy improved PFS compared with chemotherapy alone (median PFS: 6.7 vs. 6.1 months).^4^ Based on these two clinical trials, the bevacizumab combined with chemotherapy (Beva+ChT) regimen was approved by the US Food and Drug Administration (FDA) for the first‐line treatment of advanced nonsquamous NSCLC. Subsequently, the BEYOND study in China obtained similar results to the ECOG 4599 study.^5^ The Beva+ChT regimen was approved in China in 2015 for the first‐line treatment of advanced nonsquamous NSCLC.

In recent years, with the advent of immune checkpoint inhibitors (ICIs), immunotherapy plus chemotherapy (IO+ChT) regimens have rapidly become the first‐line treatment option for advanced nonsquamous NSCLC due to significantly improved OS and PFS. The results of the phase III KEYNOTE 189 study showed that pembrolizumab combined with chemotherapy had extended the overall survival time from 10.6 months to 22 months for patients with EGFR/ALK‐negative advanced nonsquamous NSCLC patients.^6^ Moreover, similar results have been confirmed by Impower130 trial, the OS (18.6 months vs. 13.9 months *p* = 0.033) and PFS (7.0 months vs. 5.5 months *p* < 0.0001) was also significantly improved in atezolizumab plus chemotherapy group.^7^ Additionally, PD‐1 inhibitors made in China, such as camrelizumab and sintilimab, have been approved in China for significantly extended survival by Camel and ORIENT‐11 trials.^8^, ^9^

However, between bevacizumab and immunotherapy, which treatment is the best partner for chemotherapy and could lead to better clinical benefits when combined with chemotherapy? No definite conclusion has yet been reached, and head‐to‐head comparative trials are lacking. Therefore, we conducted this retrospective study to evaluate the effects of Beva+ChT and IO+ChT in patients with adenocarcinoma who were negative for driver genes. Furthermore, we investigated whether the statuses of PD‐L1 expression and KRAS mutations could influence the results of the two regimens.

## 2 MATERIALS AND METHODS

### 2.1 Patients

The data of patients with advanced NSCLC who underwent Beva+ChT or IO+ChT as first‐line therapy at Shandong Cancer Hospital and Institute (Jinan, Shandong, China) between July 2018 and October 2020 were retrospectively reviewed in this study. The eligibility standards were as follows: patients with a Karnofsky performance status (KPS) score ≥ 70; patients who were histologically or cytologically diagnosed with adenocarcinoma; and patients with stage IIIB to IV NSCLC. Patients with epidermal growth factor receptor (EGFR) mutations or anaplastic lymphoma kinase (ALK) fusions were excluded from the research. Patients who did not undergo response assessments (<2 cycles or were lost to follow‐up) were also excluded. The study was approved by the Ethics Committee of Shandong Cancer Hospital and Institute. All procedures related to patients were performed according to the Declaration of Helsinki.

### 2.2 Treatments

Based on their treatment modalities, patients were classified into the Beva+ChT and IO+ChT groups. The IO+ChT group included patients treated with pembrolizumab, nivolumab, camrelizumab, and sintilimab. The patients in the Beva+ChT group included those treated with Avastin. In both cases, the ChT schemes involved pemetrexed, nab‐paclitaxel, and liposome paclitaxel. The IO+ChT or Beva+ChT regimen was repeated every 21 days for 4 to 6 cycles in total. The treatment plan for each patient was formulated according to the standard of the institution, the patient's physical state, and the attending physician's intentions.

### 2.3 Detection of PD‐L1 expression and KRAS and TP53 mutations

PD‐L1 expression was defined as the percentage of tumor cells with membranous PD‐L1‐positive staining (TPS) using 22C3 antibody by immunohistochemistry (IHC). The percentages of tumor cells with membranous PD‐L1‐positive staining (TPS) were read and recorded by three manufacturer‐trained pathologists in our pathology department; different cases were re‐examined, and consensus was reached on all cases. The PD‐L1 subgroups were defined as negative (PD‐L1 < 1%) and positive (PD‐L1 ≥ 1%). Our genotyped KRAS and TP53 mutations were evaluated by polymerase chain reaction (PCR) and amplification refractory mutation system (ARMS) using commercial kits (AmoyDx). We defined KRAS and TP53 mutations as absent (wild‐type), mutated, missing, or invalid (when insufficient tissue was available for genotyping). Missing and invalid results for the biomarkers are included in the tables.

### 2.4 Assessment of response and toxicity

Tumor response was evaluated based on the Response Evaluation Criteria in Solid Tumors (RECIST) version 1.1. After the beginning of treatment, evaluations were executed every 2 cycles and every 2 months for the next treatment session after 4–6 cycles. Adverse events (AEs) were graded based on the National Cancer Institute Common Terminology Criteria for Adverse Events (CTCAE) version 4.0.

### 2.5 Endpoints

The primary endpoint was overall survival (OS), and the secondary endpoints were progression‐free survival (PFS) and the disease control rate (DCR). OS was measured from the initiation of Beva+ChT or IO+ChT treatment to the date of death resulting from any cause, or the date of the last known follow‐up. PFS was defined as the date interval from the initiation of Beva+ChT or IO+ChT treatment to tumor progression, death resulting from any cause or the date of the last known follow‐up. According to the RECIST version 1.1, the objective response rate (ORR) incorporated the proportion of patients with a complete or partial response (CR or PR), and the DCR incorporated the proportion of patients with a CR, a PR, or stable disease (SD).

### 2.6 Statistical analysis

The comparisons of basic patient characteristics, tumor response rates, and adverse events between the two groups were analyzed by the chi‐square test and Fisher's exact test. The Kaplan–Meier method was used to analyze survival (OS, PFS) and the relevance between the clinical characteristics and survival. Multivariate survival analysis was used to evaluate the significant prognostic factors using a Cox‐proportional hazards model. The difference in survival curves between the two groups was estimated by the log‐rank test. Two‐sided *p* values <0.05 were considered statistically significant.

## 3 RESULTS

### 3.1 Patient characteristics

Between July 2018 and October 2020, 185 adenocarcinoma patients received first‐line IO+ChT or Beva+ChT treatment in our cancer center. Fifty‐four patients were excluded for possessing gene mutations (including EGFR and ALK mutations), and 26 patients were excluded for refusal of gene testing. A total of 54 patients with stage IIIB or IV NSCLC received first‐line IO+ChT treatment, and 51 received Beva+ChT treatment. The follow‐up rate was 96.2%, and four patients were lost to follow‐up. Ultimately, the study enrolled 105 patients who were treated with Beva+ChT or IO+ChT as first‐line therapy. There were 54 patients in the IO+ChT group and 51 patients in the Beva+ChT group. The last follow‐up time was April 21, 2021. Fifty‐three patients had died by the end of the follow‐up, and 52 patients were still alive. The median follow‐up time was 20.5 months (range, 6.9–42 months) for surviving patients and 16.9 months (range, 1.4–42.1 months) for all patients.

The baseline characteristics of all patients in both groups are shown in Table 1. There were no differences between the two groups in the distribution of most variables except for treatment features. The median age of the patients in the two groups was 66 years, and the age range was 36 to 76 years. Fifty‐four (51.4%) patients received first‐line IO+ChT treatment, and 51 (48.6%) patients received first‐line Beva+ChT treatment. Thirty (28.6%) patients had brain metastases, and 5 (4.8%) patients had liver metastases. Fifty‐two (49.5%) patients had never smoked. Of the 66 patients who underwent KRAS genotyping, 14 (21.2%) had KRAS mutations. Of the 31 patients who underwent TP53 genotyping, 13 (65%) patients had TP53 mutations. Fifty patients underwent PD‐L1 detection: 15 (30%) patients had a level <1%, 22 (44%) patients had a level from 1 to 49%, and 13 (26%) patients had a level ≥ 50% (Table 1).

**TABLE 1:** Patient characteristics

Characteristic	Total	IO+ChT		Beva+ChT		*p*
		No.	%	No.	%	
Gender						
Male	55	38	70.4%	17	33.3%	
Female	50	16	29.6%	34	66.7%	0.683
Age						
<65	76	38	70.4%	38	74.5%	
≥65	29	16	29.6%	13	25.5%	0.635
KPS						
<80	9	2	3.7%	7	13.7%	
≥80	96	52	96.3%	44	86.3%	0.067
Smoker						
Yes	52	24	44.4%	28	54.9%	
No	53	30	55.6%	23	45.1%	0.284
Stage						
III	15	6	11.1%	9	17.6%	
IV	90	48	88.9%	42	82.4%	0.339
KRAS mutation						
No	52	29	53.7%	23	45.1%	
Yes	21	11	20.4%	10	19.6%	
Undetected	32	14	25.9%	18	35.3%	0.561
TP53 mutation						
No	7	2	3.7%	5	9.8%	
Yes	13	7	13.0%	6	11.8%	
Undetected	85	45	83.3%	40	78.4%	0.456
Brain meta						
Yes	30	14	25.9%	16	31.4%	
No	75	40	74.1%	35	68.6%	0.537
Liver meta						
Yes	5	2	3.7%	3	5.9%	
No	100	52	96.3%	48	94.1%	0.600
PD‐L1 TPS						
<1%	33	16	29.6%	17	33.3%	
≥1%	39	25	46.3%	14	27.5%	
Undetected	33	13	24.1%	20	39.2%	0.104

### 3.2 Responses

Among all 105 patients, the ORR was 35.2%, and the DCR was 84.8%. The ORR was 40.7% in the IO+ChT group and 29.4% in the Beva+ChT group (*p* = 0.043). The DCR was 84.7% in the IO+ChT group and 84.3% in the Beva+ChT group (*p* = 0.185). The difference in the response rate between the two groups was significant (Table 2).

**TABLE 2:** Tumor response

	IO+CHT (n = 54)	Beva+CHT (n = 51)
Objective response rate, *n* (%)	29 (53.7%)	16 (31.4%)
*p* value	0.021	
Disease control rate, *n* (%)	46 (85.2%)	42 (82.4%)
*p* value	0.694	
Breast overall response, *n* (%)		
Complete response	0	0
Partial response	29 (53.7%)	16 (31.4%)
Stable response	17 (31.5%)	26 (51.0%)
Progressive disease	8 (14.8%)	9 (17.6%)

### 3.3 Survival

**FIGURE 1:**
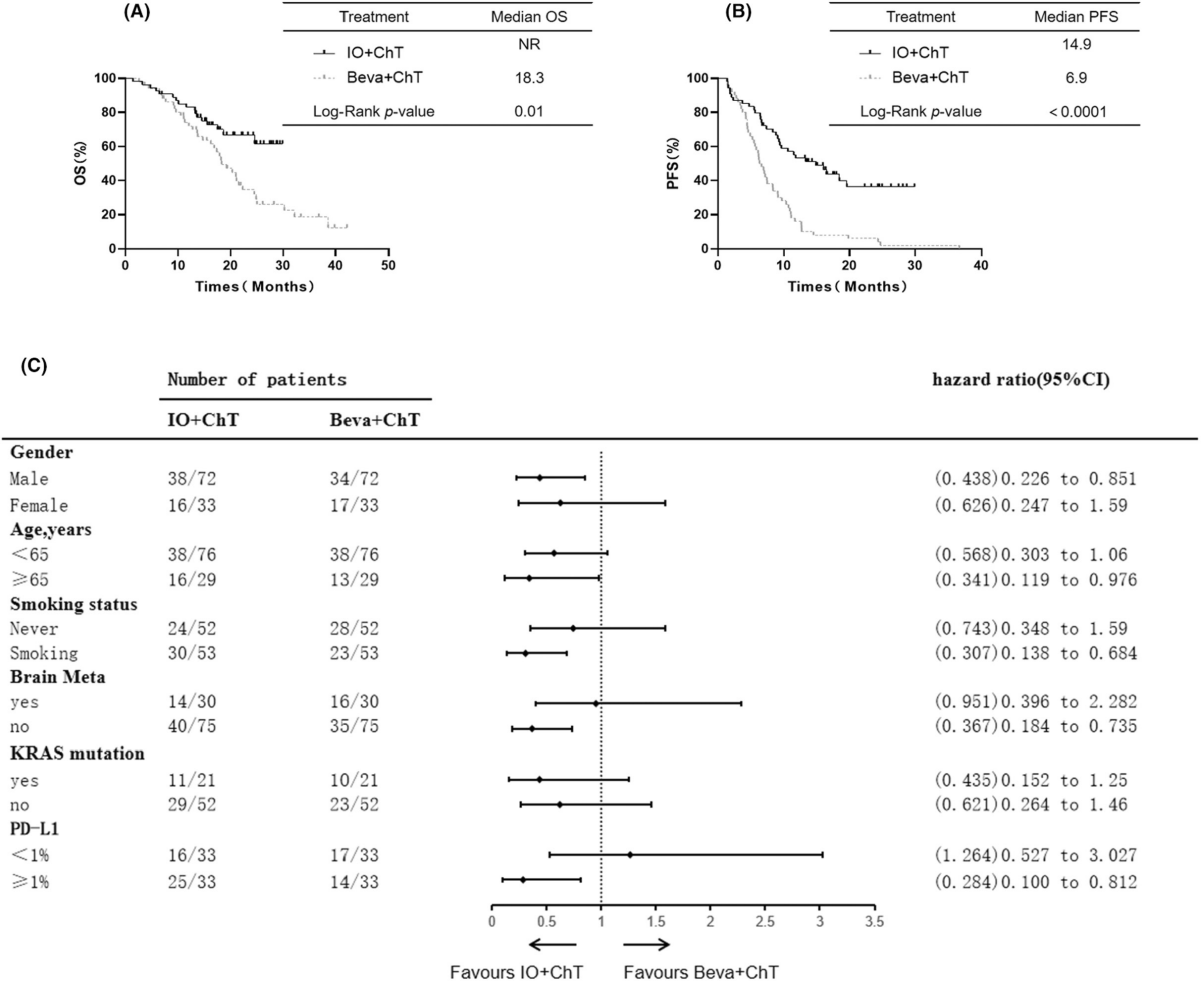
IO+ChT versus Beva+ChT in first‐line treatment of OS (A) and PFS (B) in advanced adenocarcinoma NSCLC patients. Subgroup analysis of OS (C). Abbreviations: Beva, bevacizumab; ChT, chemotherapy; IO, immunotherapy; NSCLC, non‐small‐cell lung cancer; OS, overall survival; PFS, progression‐free survival.

The median OS in the IO+ChT group and Beva+ChT group was NR and 18.3 months, respectively. The OS rates at 1 year in the IO+ChT group and Beva+ChT group were 100% and 93.8%, respectively. The OS was longer in the IO+ChT group than in the Beva+ChT group among all 105 patients (*p* = 0.01, Figure 1A). The median PFS was 14.9 months for the patients receiving IO+ChT treatment and 6.3 months for those receiving Beva+ChT treatment. The PFS was longer in the IO+ChT group than in the Beva+ChT group (*p* < 0.001, Figure 1B). The PFS rates at 1 year in the IO+ChT group and Beva+ChT group were 48.2% and 20.8%, respectively. Partial subgroup analysis of OS was in Figure 1C.

**FIGURE 2:**
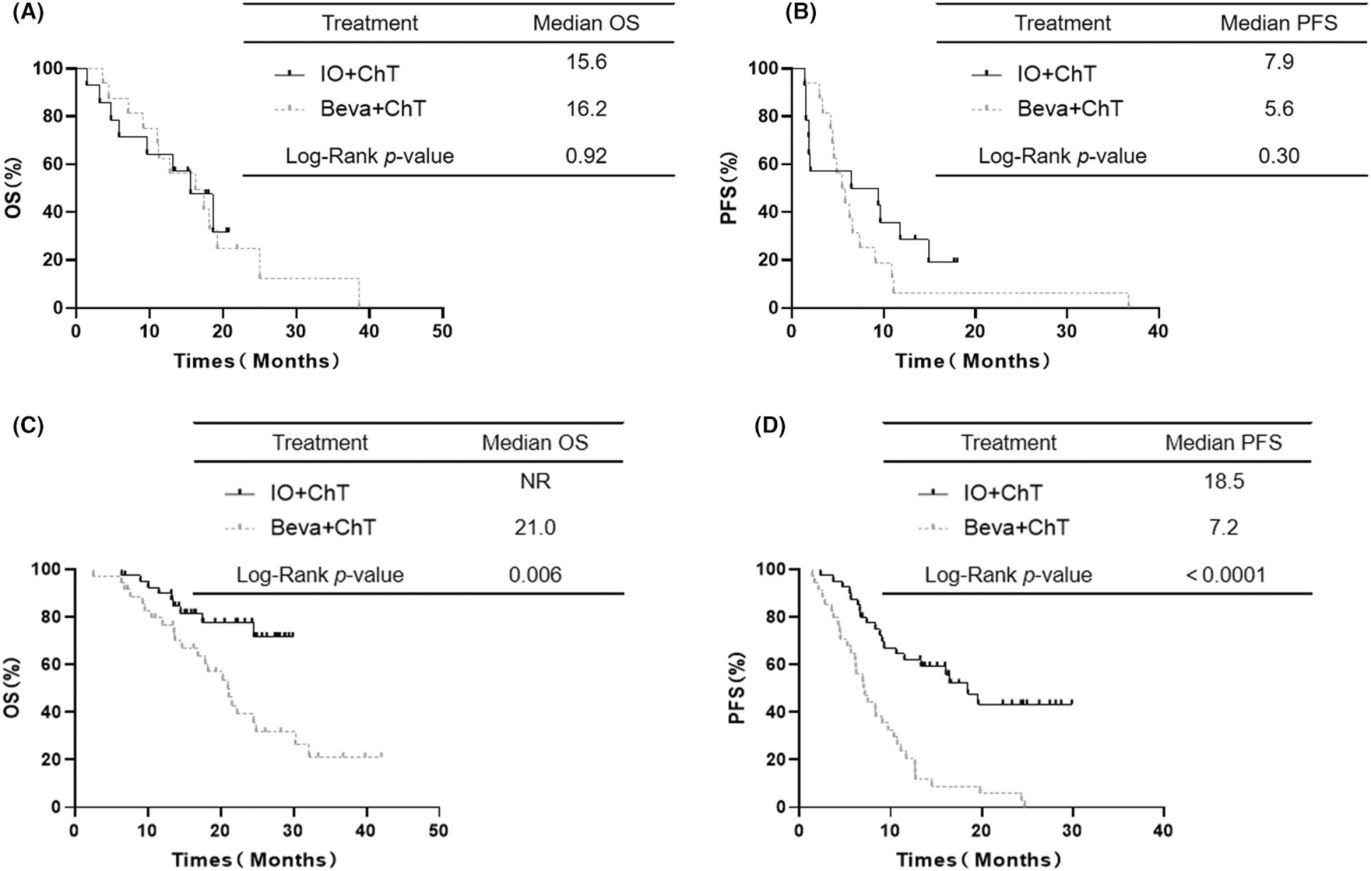
OS (A) and PFS (B) in the BM subgroup of advanced adenocarcinoma NSCLC with first‐line therapy between IO+ChT and Beva+ChT. OS (C) and PFS (D) in no BM subgroup of advanced adenocarcinoma NSCLC with first‐line therapy between IO+ChT and Beva+ChT.

In the subgroup of 30 patients with brain metastases, the OS and PFS were not significantly different between the IO+ChT group and the Beva+ChT group (median OS: 15.6 vs. 16.2 months, respectively, *p* = 0.91 Figure 2A; median PFS: 6.5 vs. 5.5 months, respectively, *p* = 0.30, Figure 2B). Moreover, in the 75 patients without brain metastases, the OS and PFS (median OS: NR vs. 21.0 months, *p* = 0.006 Figure 2C; median PFS: 18.5 vs. 7.2 months, *p* < 0.0001, Figure 2D) were longer in the IO+ChT group than in the Beva+ChT group.

**FIGURE 3:**
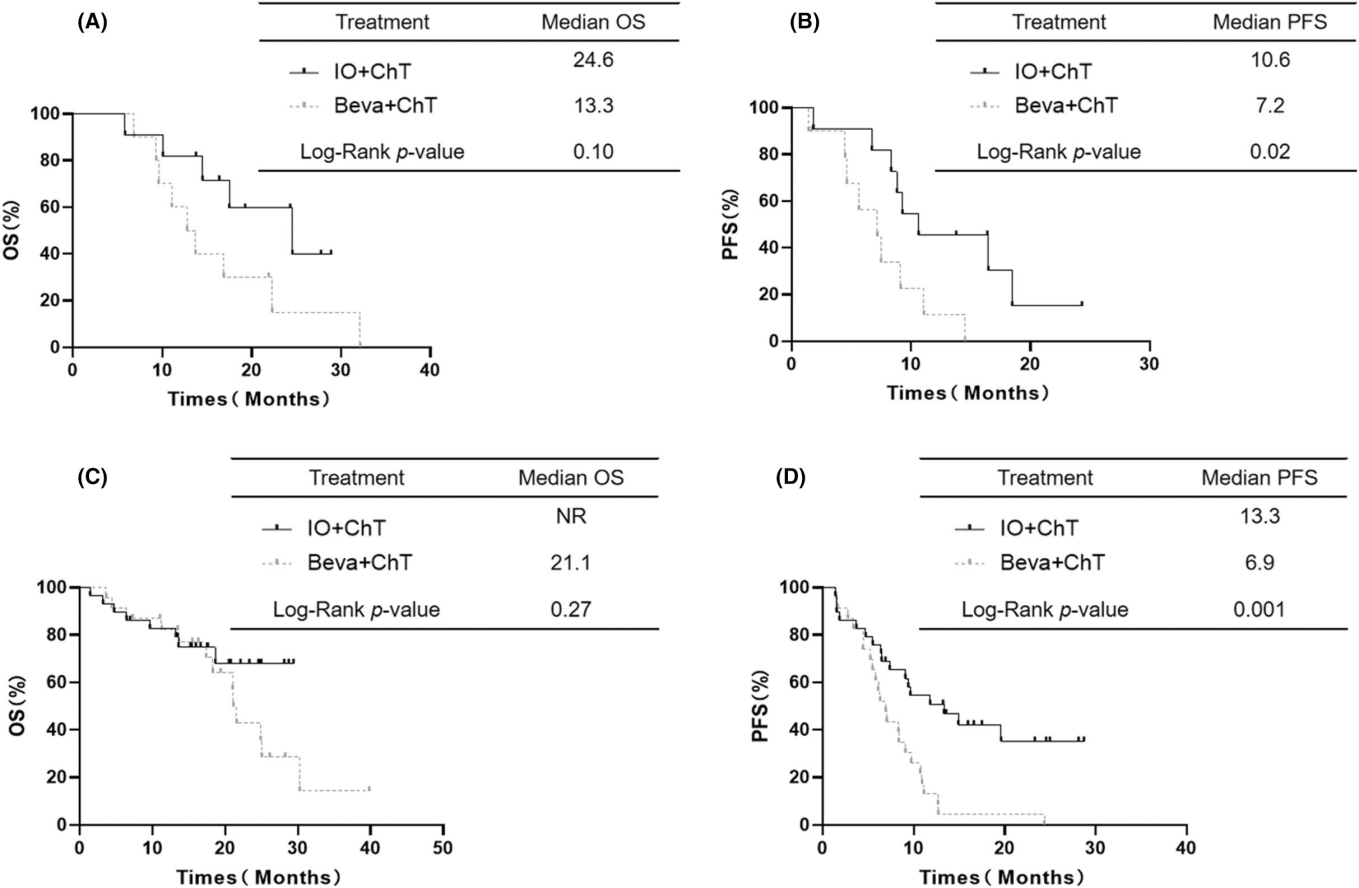
OS (A) and PFS (B) in the KRAS mutation subgroup treated with first‐line therapy between IO+ChT and Beva+ChT. OS (C) and PFS (D) in patients without KRAS mutation subgroup treated with first‐line therapy between IO+ChT and Beva+ChT.

In the subgroup of the 21 patients with a KRAS mutation (MT), the OS was not significantly different between the IO+ChT group and the Beva+ChT group (median OS: 24.6 vs. 13.3 months, respectively, *p* = 0.10, Figure 3A). The PFS in the IO+ChT group was longer than that in the Beva+ChT group (median PFS: 10.6 vs. 7.2 months, respectively, *p* = 0.02, Figure 3B). In the subgroup of the 52 patients without a KRAS mutation, the PFS was also longer in the IO+ChT group than in the Beva+ChT group (median PFS: 13.3 vs. 6.9 months, respectively, *p* = 0.001, Figure 3D). The OS was not different between the IO+ChT group and the Beva+ChT group (median OS: NR vs. 21.1 months, respectively, *p* = 0.27, Figure 3C). In the ICI plus ChT group, there was no difference in OS (*p* = 0.51) or PFS (*p* = 0.69) between the patients with a KRAS MT and those with a KRAS MT. Similarly, no difference was found in OS (*p* = 0.15) or PFS (*p* = 0.97) in the Beva plus ChT group.

**FIGURE 4:**
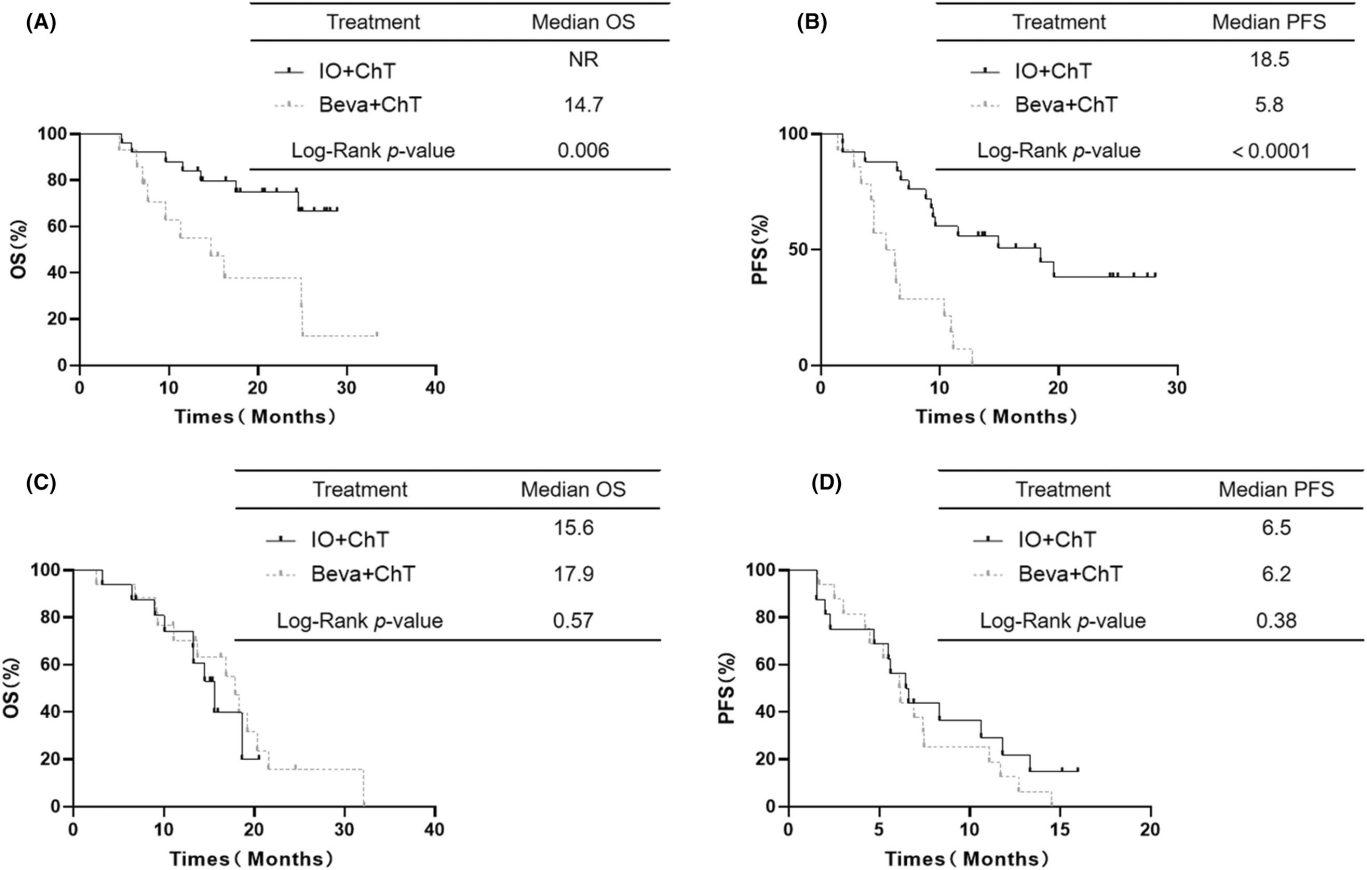
OS (A) and PFS (B) in PD‐L1 ≥ 1% subgroup between IO+ChT and Beva+ChT. OS(C) and PFS (D) in PD‐L1<1% subgroup between IO+ChT and Beva + ChT.

The IO+ChT group showed a longer OS (median OS: NR vs. 14.7 months, respectively, *p* = 0.006, Figure 4A) and PFS (median PFS: 18.5 vs. 5.8 months, respectively, *p* < 0.0001, Figure 4B) than the Beva+ChT group for PD‐L1 positive (≥1%) subgroups. However, the OS (median OS: 15.6 vs. 17.9 months, respectively, *p* = 0.57, Figure 4C) and PFS (median PFS: 6.5 vs. 6.2 months, respectively, *p* = 0.38, Figure 4D) were not different between the IO+ChT group and the Beva+ChT group in the PD‐L1‐negative (<1%) subgroups.

### 3.4 Prognostic factors

The clinical characteristics of the patients were evaluated to determine their prognostic value for OS (Table 3). Univariate analysis indicated that brain metastases and treatment were associated with survival. Patients without brain metastases had a better OS than patients with brain metastases (*p* = 0.001). Moreover, multivariate analyses indicated that brain metastases were also associated with OS. For PFS, univariate analysis revealed that brain metastasis was a significant favorable prognostic factor (Table 4). Multivariate analysis revealed that the KPS score (*p* = 0.019), brain metastasis (*p* = 0.014) and radiotherapy (*p* = 0.045) were favorable prognostic factors for PFS.

**TABLE 3:** Univariate analysis and multivariate analysis for OS

	Univariate analysis		Multivariate analysis	
Factors	HR (95%CI)	*p*	HR (95%CI)	*p*
Gender (male/female)	1.248 (0.705–2.209)	0.448		
Age (<65/≥65)	1.084 (0.584–2.013)	0.798		
KPS (<80/≥80)	0.654 (0.290–1.475)	0.306		
Smoking status (never/smoking)	0.937 (0.545–1.611)	0.815		
Brain meta (no/yes)	2.557 (1.457–4.489)	0.019	2.320 (1.195–4.506)	0.013
Liver meta (no/yes)	0.583 (0.141–2.407)	0.456		
KRAS mutation (no/yes)	1.606 (0.815–3.167)	0.171		
PD‐L1(<1%/≥1%)	0.462 (0.241–0.888)	0.021	0.441 (0.229–0.850)	0.014

**TABLE 4:** Univariate analysis and multivariate analysis for PFS

	Univariate analysis		Multivariate analysis	
Factors	HR (95%CI)	*p*	HR (95%CI)	*p*
Gender (male/female)	1.342 (0.843–2.138)	0.215		
Age (<65/≥65)	0.719 (0.429–1.204)	0.210		
KPS (<80/≥80)	0.913 (0.438–1.902)	0.808		
Smoking status (never/smoking)	0.670 (0.431–1.044)	0.077		
Stage (III/IV)	0.961 (0.519–1.778)	0.899		
Brain meta (no/yes)	1.920 (1.197–3.080)	0.009	2.205 (1.264–3.847)	0.005
Liver meta (no/yes)	0.650 (0.237–1.784)	0.403		
KRAS mutation (no/yes)	1.107 (0.625–1.959)	0.728		
PD‐L1 (<1%/≥1%)	0.507 (0.296–0.867)	0.013	0.483 (0.282–0.827)	0.008

### 3.5 Toxicities

Table 5 lists rates of adverse events (AEs) in two treatment groups. The rate of any grade AEs in IO+ChT and Beva+ ChT groups was 24.1% and 27.5%, respectively. The incidence of grade ≥3 AEs occurred with a higher frequency in Beva+ ChT group (9.8%) compared with IO+ChT group (3.7%), but no statistical difference (*p* = 0.651). One patient in each group stopped taking drugs because of neutrophil decreased and one patient because of diarrhea. No patient died from AEs.

**TABLE 5:** Adverse events of any cause

Treatment‐related AEs, *n* (%)	IO+ChT (*n* = 54)	Beva+ChT (n = 51)	*p*
Any grade	13 (24.1%)	14 (27.5%)	
Decreased neutrophil	4 (7.4%)	7 (9.8%)	
Anemia	2 (3.7%)	1 (2.0%)	
Diarrhea	1 (1.9%)	1 (2.0%)	
Vomiting	0 (0%)	3 (5.9%)	
Fatigue	1 (1.9%)	1 (2.0%)	
Pneumonitis	2 (3.7%)	0 (0%)	
Increased aspartate aminotransferase	2 (3.7%)	1 (2.0%)	
Hypothyroidism	1 (1.9%)	0 (0%)	
Myocarditis	2 (3.7%)	0 (0%)	
Rash	0 (0%)	1 (2.0%)	
Hypolbuminemia	0 (0%)	1 (2.0%)	
Grade ≥3	3 (3.7%)	5 (9.8%)	0.651
Decreased neutrophil	3 (3.7%)	4 (7.8%)	
Diarrhea	0 (0%)	1 (2.0%)	
AEs leading to discontinuation	1 (1.9%)	2 (3.9%)	
Diarrhea	0 (0%)	1 (2.0%)	
Decreased neutrophil	1 (1.9%)	1 (2.0%)	
AEs leading to death	0 (0%)	0 (0%)	

## 4 DISCUSSION

With the identification of many new targets, a variety of options, such as antiangiogenic drugs and immune checkpoint inhibitors, have become available for nonsquamous cell NSCLC patients without driver gene alterations. The ECOG 4599 study demonstrated improved OS (12.3 months vs. 10.3 months, HR = 0.79, *p* = 0.003) and PFS (6.2 months vs. 4.5 months, HR = 0.66, *p* < 0.001) for bevacizumab combined with chemotherapy versus chemotherapy alone. A similar result was observed in the BEYOND trials, which allowed bevacizumab plus chemotherapy for the treatment of nonsquamous cell lung cancer. In the era of immunotherapy, the KEYNOTE 189, Impower130, Camel, and ORIENT‐11 trials revealed superior benefits of IO+ChT treatment over chemotherapy alone, regardless of PD‐L1 status.^6^, ^7^, ^8^, ^9^ However, the best treatment choice and how to choose the combination therapy for this population remain inconclusive. In our study, a comparison of IO+ChT and Beva+ChT treatment revealed significant differences in OS and PFS for nonsquamous cell (NSCLC) patients without driver gene alterations. Specifically, the OS and PFS in the IO+ChT group were longer than those in the Beva+ChT group. Remarkably, our median PFS was 6.3 m in Beva+ChT group, was somewhere between 9.2 m and 6.2 m of B + CP arms in the BEYOND and ECOG4599 study. Patients with EGFR mutations excluded from our study but 23% of EGFR mutation included in B + CP arms in the BEYOND study. Researches^27^, ^28^ have shown improved OS and PFS with the addition of Beva in EGFR‐mutations. Additionally, CNS metastases have been excluded in the BEYOND study while our study not, which is also considered as one of the reasons for the relatively low PFS in the Beva group.

Univariate and multivariate analyses suggested that no brain metastases (BMs) and PD‐L1 levels ≥1% were favorable factors for OS and PFS. Approximately 26% of advanced NSCLC patients have brain metastasis (BM) at diagnosis, which indicates a poor prognosis.^10^ However, scarce data are available, as BMs have always been exclusion criteria in previous trials. Several trials have revealed benefits for BM, oncogene‐driven NSCLC patients treated with targeted drugs,^11^, ^12^ while scarce data are available for patients without oncogenic driver genes. An updated analysis of the Keynote‐189 trial data reported a median OS of 22.0 months and a PFS of 11.1 months for 35 nonsquamous BM NSCLC patients without sensitizing EGFR/ALK alterations while excluding symptomatic patients with CNS metastasis. The bevacizumab combined group showed improved OS and PFS in the ECOG 4599 and BEYOND trials.^3^, ^5^ The median OS was 12.3 and 24.3 months, and the median PFS was 6.2 and 9.2 months, respectively. Our subgroup of 30 BM NSCLC patients without oncogenic driver genes was treated with IO or bevacizumab combined with chemotherapy. The OS and PFS showed no statistically significant difference between the two groups. The overall median OS was 16.2 months, while in the IO+ChT and Beva+ChT groups, the median OS was 15.6 and 16.2 months, respectively (*p* = 0.907). In addition, the median PFS was 6.5 months versus 5.5 months, respectively (*p* = 0.304). Compared with previous clinical trials, our shorter OS and PFS might have been due to BM patients with symptomatic CNS metastases not being excluded in our study. The brain was previously considered an immune‐exempt organ, with the blood–brain barrier thought to be impenetrable. However, BM patients with a changed tumor microenvironment (TME) have shown benefits via multiple mechanisms reported for either Beva or ICIs combined with chemotherapy.^13^, ^14^, ^15^ The Impower150 trial showed prolonged OS and PFS in the atezolizumab + Beva + CHT group compared with either the atezolizumab + ChT or Beva + ChT group, while related data for BM patients were absent. In the 2020 ESMO study, nivolumab in combination with chemotherapy and bevacizumab showed superior PFS for patients with advanced or recurrent nonsquamous NSCLC than for those without the addition of nivolumab (HR = 0.65; 95% CI: 0.36–1.18) in an interim phase 3 trial. Collectively, BM patients with lung adenocarcinoma (LADC) without oncogenic drivers benefit from multiple regimens. Further studies are required to evaluate the role of these combination regimens in the prevention and treatment of brain metastases.

Programmed death‐ligand 1 (PD‐L1) is an immune regulator that induces immunosuppression by binding to the programmed death‐1 receptor of T lymphocytes. High PD‐L1 expression was reported to be highly associated with poor prognosis in several studies.^16^, ^17^ Despite the lack of sufficiently randomized studies, PD‐L1 expression is currently considered the most promising predictive marker. The Keynote‐189 trial reported a significant OS benefit (22 months vs. 10.6 months, respectively) of pembrolizumab combined with chemotherapy (PC) compared with chemotherapy alone as the first‐line therapy in nonsquamous cell NSCLC regardless of PD‐L1 status^6^ A network meta‐analysis based on six separate trials with metastatic nonsquamous cell NSCLC suggested that PC performed better in terms of OS (HR 0.38, 95% CI 0.16–0.87) and PFS (HR 0.45, 95% CI 0.24–0.86) in a population with high PD‐L1 expression (>50%), but no significant difference was observed in a population with moderate expression (1–49%), and a better prognosis was still observed in a population with positive (<1%) expression.^18^ Consistent with previous results, our results showed a significant difference in OS and PFS in the PD‐L1‐positive (PD‐L1 ≥ 1%) subgroup but showed no difference in the PD‐L1‐negative (PD‐L1 < 1%) subgroup. The difference in our results might be related to the inclusion of all types of ICIs in our study. PD‐1 and PD‐L1 inhibitors did not result in the same survival benefit or immune‐related events (irAEs) due to differences in the receptors.

With an approximately 30% mutation rate, KRAS is the most commonly mutated gene in NSCLC.^19^ Studies have reported a significantly increased mutation burden and poor prognosis in NSCLC patients with KRAS mutations (MTs). To date, no targeted drugs have shown efficacy against KRAS, and platinum‐based treatment is still the standard first‐line therapy. ICIs have shown conflicting results as second‐line and rear‐line treatments. The Checkmate 057 trials showed a longer OS benefit from nivolumab in the KRAS MT subgroup (HR 0.52, 95% CI 0.29–0.95).^20^ A meta‐analysis including 138 NSCLC patients with KRAS MT and 371 with KRAS wild‐type (WT) MTs reported that ICIs improved OS in the KRAS MT cohort compared with docetaxel (HR = 0.64 [95% CI, 0.43–0.96], *p* = 0.03), while the KRAS wild‐type cohort showed no difference.^21^ In addition, a study of 530 pretreated advanced nonsquamous cell NSCLC patients treated with nivolumab reported that only the 3‐month PFS rate was significantly higher in the KRAS MT group than in the KRAS WT group, while the OS (4 months vs. 3 months, *p* = 0.5) and PFS were not significantly different (11.2 months vs. 10 months, *p* = 0.8).^20^ Only a few studies have investigated the relevance of Beva therapy in NSCLC. Anna et al. showed no statistically significant difference between stage IV adenocarcinoma patients with KRAS MTs and KRAS WT treated with first‐line Beva/CHT in terms of OS (HR 1.12, 95% CI 0.58–2.16, *p* = 0.74) or PFS (HR 0.80, 95% CI 0.48–1.34, *p* = 0.41).^22^ Ghimessy et al. reported a negative relationship between KRAS MTs and prognosis in stage IIIB‐IV adenocarcinoma NSCLC patients treated with Beva plus CHT. KRAS MT patients also showed a shorter OS (p = 0.0186) and PFS (*p* = 0.0255).^23^ Patients with KRAS MTs do not seem to benefit from Beva therapy. Of the 105 patients involved in our study, 71 patients underwent KRAS genotyping, and the KRAS‐positive rate was 28.8%, which was lower than that in most trials. Compared with the Beva combined group, we found that the ICT plus CHT group received a statistically significant PFS benefit in both the KRAS mutation (*p* = 0.022) and KRAS wild‐type groups (*p* = 0.018) compared with the Beva + ChT group, while there was no statistically significant difference in OS. There was no significant difference in OS (*p* = 0.507) or PFS (*p* = 0.688) between KRAS MT and KRAS WT patients treated with IO plus ChT. Similarly, no difference was found in the Beva plus ChT group, consistent with the results of previous experiments. Notably, KRAS mutations were demonstrated to be accompanied by high PD‐L1 expression^24^, ^25^ and may be related to the ICI treatment effect. Due to the lower KRAS mutation rate relative to several trials, our OS and PFS require further testing with a larger sample.

TP53 is another commonly mutated gene in LADC. TP53, KRAS, and combined mutations have all been reported to be associated with high PD‐L1 expression. Combined TP53 and KRAS mutations have also been reported to be associated with longer survival.^26^ Only five patients in our study had combined mutations, and further analysis was not conducted.

The rate of any grade AEs was basically common in IO+ChT group and Beva+ChT group (24.1% vs 27.5%). Although Grade ≥3 AEs with a higher rate in Beva+ChT group, but there was no statistical difference between the two groups. AEs of any grade in our study were underestimated in our study, probably because low‐grade adverse reactions were easily ignored by patients and not actively reported.

In our study, the incidence of pneumonitis in the IO+ChT group was 3.7%, in agreement with ORIENT‐11 and KEYNNOTE‐189 trials (3.4%–4.4%). The most common AEs in our study were neutrophil decreased whatever in any grade or in Grade ≥3 AEs, which was consistent in IO and Beva related trials.^3^, ^4^, ^5^, ^6^, ^7^, ^8^, ^9^ Neutrophil decreased should be taken seriously and enhancement of monitoring in treatment.

Like all retrospective analyses, our study has limitations. First, the small sample size affected the statistical power and may have led to selection and measurement bias. Despite adjustment by the Cox regression model, confounding factors may still have been present. Further analysis with a larger sample size is necessary in the future. Second, the heterogeneity of the specimens and different visual evaluations by pathologists might have led to biased results. Moreover, the previously reported KRAS mutation rate was approximately 30%, while our rate of KRAS mutations was 19.4%, and we did not perform further typing due to the small sample size. However, a study reported that the KRAS G12C cohort had significantly better OS and PFS. Finally, the available baseline features in our retrospective study were limited. Some important clinical information that may affect the survival time and treatment response, such as the PD‐L1 tumor proportion score or TMB level and baseline lung function, was not available for all patients.

## 5 CONCLUSION

In conclusion, our study suggested that ICIs combined with ChT improved clinical outcomes compared with Beva combined with ChT as the first‐line therapy in adenocarcinoma patients without driver gene alterations. Our study tried to demonstrate cohort benefits in treatment selection. Compared to the Beva combined ChT group, patients treated with ICIs combined with ChT showed benefits in the PD‐L1 ≥ 1% subgroup, but patients with BM, KRAS mutations, and PD‐L1 levels <1% did not show further benefits in our study. Our observations might provide a way to combine predictors for treatments. Larger trials are needed for further confirmation.

## AUTHOR CONTRIBUTIONS

**Min Wang:** Writing – original draft (lead). **Ji Li:** Resources (supporting). **Shuhui Xu:** Data curation (supporting). **Yuying Li:** Data curation (supporting). **Jiatong Li:** Data curation (supporting). **Jinming Yu:** Writing – review and editing (equal). **Xiaoyong Tang:** Writing – review and editing (equal). **Hui Zhu:** Writing – review and editing (equal).

## FUNDING INFORMATION

This work was supported by CSCO‐Pilot Cancer Research Fund [grant number: Y‐2019AZZD‐0352], Key Research and Development Program of Shandong Province [grant number: 2018GSF118067], and Natural Science Foundation of Shandong Province (ZR2019LZL018).

## DISCLOSURE

The authors declare that the research was conducted in the absence of any commercial or financial relationships that could be construed as a potential conflict of interest.

## Data Availability

The data that support the findings of this study are available from the corresponding author upon reasonable request
